# Interrupted coding sequences in *Mycobacterium smegmatis*: authentic mutations or sequencing errors?

**DOI:** 10.1186/gb-2007-8-2-r20

**Published:** 2007-02-12

**Authors:** Caroline Deshayes, Emmanuel Perrodou, Sebastien Gallien, Daniel Euphrasie, Christine Schaeffer, Alain Van-Dorsselaer, Olivier Poch, Odile Lecompte, Jean-Marc Reyrat

**Affiliations:** 1 Université Paris Descartes, Faculté de Médecine René Descartes, Paris Cedex 15, F-75730, France; 2 Inserm, U570, Unité de Pathogénie des Infections Systémiques-Groupe AVENIR, Paris Cedex 15, F-75730, France; 3 Laboratoire de Biologie et Génomique Structurales, IGBMC CNRS/INSERM/ULP, BP 163, 67404 Illkirch Cedex, France; 4 Laboratoire de Spectrométrie de Masse Bio-Organique, UMR7178, ECPM, rue Becquerel, Strasbourg, F-67087 cedex 2, France

## Abstract

**Background:**

*In silico *analysis has shown that all bacterial genomes contain a low percentage of ORFs with undetected frameshifts and in-frame stop codons. These interrupted coding sequences (ICDSs) may really be present in the organism or may result from misannotation based on sequencing errors. The reality or otherwise of these sequences has major implications for all subsequent functional characterization steps, including module prediction, comparative genomics and high-throughput proteomic projects.

**Results:**

We show here, using *Mycobacterium smegmatis *as a model species, that a significant proportion of these ICDSs result from sequencing errors. We used a resequencing procedure and mass spectrometry analysis to determine the nature of a number of ICDSs in this organism. We found that 28 of the 73 ICDSs investigated correspond to sequencing errors.

**Conclusion:**

The correction of these errors results in modification of the predicted amino acid sequences of the corresponding proteins and changes in annotation. We suggest that each bacterial ICDS should be investigated individually, to determine its true status and to ensure that the genome sequence is appropriate for comparative genomics analyses.

## Abstract 2

The question of whether bacterial interrupted coding sequences (ICDS) should be individually verified to produce an informative genome sequence is raised after bioinformatic, proteomic and sequencing analyses reveal that a significant proportion of ICDSs in the deposited genome sequence of *Mycobacterium smegmatis *are a result of sequencing errors.

## Background

More than 250 complete bacterial genome sequences are now available, providing unprecedented opportunities for investigating gene and protein functions [1]. The introduction of errors at the first stage of genome sequencing and gene prediction has a major impact on all subsequent studies. One source of errors in genome annotation is the sequence itself. The development of programs identifying position-specific errors has considerably increased the quality of genomic sequences [2-4]. These errors may introduce stop codons or 'artificial' frameshifts in the coding region that are easily detected by computer-assisted methods [5-7]. Such sequence errors lead to errors in annotation and comparison. An *in silico *survey of the published bacterial genomes shows that most contain interrupted coding sequences (ICDSs) [5-7]. They occur at low frequency, between 2 and 258 per Mb, not correlated with the size or GC content of the genome. A mean of 74 ICDSs were identified per prokaryotic genome tested [5]. If this is translated into ICDSs per total coding sequences, a figure of 1% to 5% is obtained, with similar figures reported by various independent studies [5,8]. The only notable exception is *Mycobacterium leprae*, which has 30% ICDSs, frequently described as pseudogenes [8]. ICDSs may be present in genes of known or unknown function. A number of bacterial species are known to have developed sophisticated mechanisms for bypassing frameshifts and restoring the correct reading frame, but such mechanisms are unlikely to be general [9,10]. Moreover, the frameshifts bypassed by the ribosome are generally preceded by a unique sequence that can be identified [11]. Thus, the detected ICDSs may either reflect the real genome sequence of the organism, with all the ensuing consequences for the composition of the encoded protein, or they may result from sequencing errors.

We used *M. smegmatis *mc^2^155 as the model species for this study. This saprophytic bacterium, which is often used as a model organism for studies of *M. tuberculosis *functions, has recently been sequenced [12]. By resequencing the ICDSs of this strain, we show that the genome sequence of this organism contains multiple errors. We systematically corrected the errors, and in all cases, these corrections rendered the predicted protein more similar to its ortholog. We also confirm, by a combined proteome and mass spectrometry analysis, that the sequences of some proteins have been incorrectly predicted due to sequencing errors. However, several ICDSs do correspond to true frameshifts. Authentic frameshifts provide a positive addition to our knowledge and make it possible to investigate gene and protein function, whereas sequencing errors generate false knowledge and confound comparative analyses. We show here that the individual analysis of ICDSs can lead to re-evaluation of the annotation of the genome and the proteome. We suggest that each bacterial ICDS should be investigated individually to ascertain its status and to produce a genome sequence suitable for productive comparative genomics.

## Results

### ICDSs in *M. smegmatis *mc^2^155: a resequencing analysis

An *in silico *analysis of the genome of *M. smegmatis *mc^2^155 revealed that it contains 94 ICDSs [5]. The ICDS database was created using a program based on the analysis of physically adjacent genes to predict putative ICDSs in complete genomes. Briefly, pairs of adjacent genes with at least one common homolog are defined as 'coding sequences (CDSs) containing common hits' and may correspond to a pair of adjacent paralogs or ICDSs. We excluded paralogs from the analysis by searching for sequence similarity between the two 'CDSs containing common hits'. The remaining CDSs are considered to be ICDSs, indicating frameshifts or in-frame stop codon insertion, due to sequencing errors or authentic events. These 94 ICDSs account for 1.4% of the total coding capacity of this organism. They may result from mutations acquired during evolution or from errors in genome sequencing.

**Figure 1:**
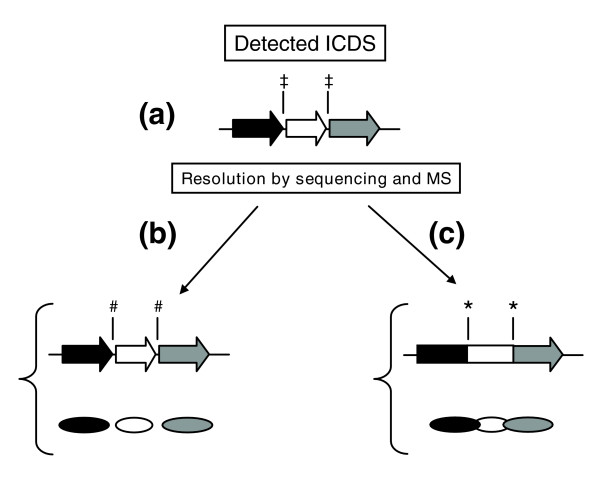
Scheme for ICDS detection and resolution strategy. **(a) **ICDSs are detected within the genome by *in silico *analysis. The double daggers (‡) indicate the regions containing the identified frameshift. Upon resolution by sequencing and mass spectrometry analysis, the ICDSs can be classified as **(b) **true frameshifts or **(c) **sequencing errors. The hash symbol (#) indicates the region of the ORF containing the frameshift. The asterisks (*) indicate sites of corrected sequencing errors resulting in the reconstitution of a full-length ORF. The ORFs are depicted with arrows. The ORF may or may not be in the same frame. Proteins are represented by ellipses.

We resequenced the genome of this strain to determine the status of these ICDSs. We did not resequence 21 ICDSs due to the duplication of some open reading frames (ORFs) or high levels of paralogy. The remaining 73 ICDSs were amplified and sequenced on both strands. We compared the nucleotide sequences obtained with the publicly available genome sequence of *M. smegmatis *mc^2^155. We found that 28 of the 73 ICDSs investigated correspond to sequencing errors (Table 1). These 28 genes containing sequencing errors correspond to 4 errors per megabase in the complete genome. In most cases, correction of the error reunified two adjacent ORFs, resulting in a single ORF rather than the two small ORFs of the original sequence (Figure 1).

Three types of error can be distinguished: miscall, overcall and undercall (Table 1) [2-4]. However, no miscalls (incorrect prediction of a specific nucleotide at a given position) were observed within the 28 sequences containing errors, due to the nature of the program used. The predicted amino acid sequences derived from the corrected nucleotide sequences differed greatly from the original predicted sequences and, in all cases, were systematically more similar to their orthologs. In one case (ICDS0089), the ORF containing the frameshift was not even predicted; the frameshift was probably responsible for the non-assignment of this ORF. The genes affected by the sequencing errors encode proteins of several classes, including 'unknown', 'intermediary metabolism', 'regulation' and 'lipid metabolism' (Table 1). The genes containing frameshifts encode proteins of several classes, including all of those cited above (Table 2). No particular pattern of nucleotides was associated with the 28 sequences containing errors or with the 45 sequences containing frameshifts.

As *M. smegmatis *mc^2^155 was derived from strain ATCC607, we carried out a comparative analysis of the ICDSs in these two strains. The mc^2^155 strain was generated from ATCC607 by selection for adaptation to genetic manipulation [13]. The mc^2^155 strain differs phenotypically from its progenitor (ATCC607) in several ways [13,14]. The frameshifts in mc^2^155 may well have been acquired recently in the laboratory, due either to counter-selection of pathways of little utility or selection for genetic manipulability. We therefore investigated whether the genes containing frameshifts were acquired before or after the divergence of the two strains. The genome of the ATCC607 strain has not been sequenced, but as both strains belong to the same species (*M. smegmatis*), the sequencing primers originally designed for the mc^2^155 strain could also be used for the ATCC607 strain. We resequenced the 45 genes containing a frameshift of mc^2^155 strain in ATCC607 (Table 2). All these genes but one (ICDS0020) also contain a frameshift in the progenitor (ATCC607), suggesting that these mutations were acquired before the divergence of the two strains. Thus, the selection of the mc^2^155 strain and its repeated culture in laboratory conditions had no major impact on frameshift acquisition and pseudogene formation.

Our analysis shows that the genome sequence of *M. smegmatis *mc^2^155 contains ICDSs, some of which correspond to authentic mutations acquired during evolution, with others resulting entirely from sequencing errors. Our results show that 18 predicted genes do not actually exist in this species (due to fusion of the two ORFs following the correction of the errors) and that one gene was even not predicted in the former sequence, presumably due to these sequencing errors. In all cases, the new predicted genes are actually more similar than previously thought to orthologs in other species.

### ICDSs in *M. smegmatis *mc^2^155: a proteome analysis

**Figure 2:**
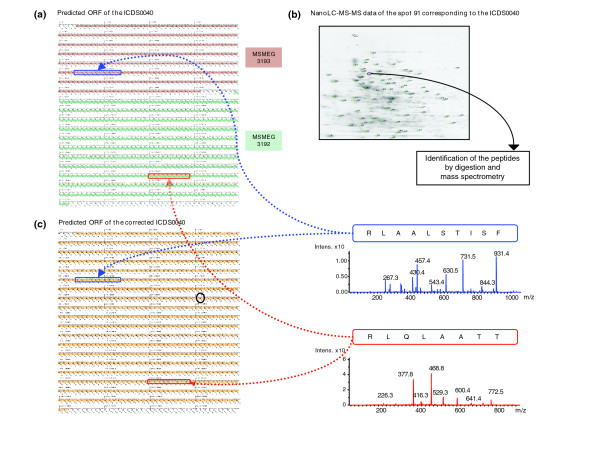
Comparison of genomic prediction with proteomic results (example of ICDS0040). **(a) **Representation of the DNA region and its predicted ORFs (in color). **(b) **Detailed view of the two-dimensional gel. Nano-LC-MS-MS data are obtained after extraction and digestion of the protein. The matching peptides are boxed in the translated genomic sequence (a,c). **(c) **Representation of the DNA region and its predicted ORF upon correction of the sequencing errors (depicted in the ellipse). Correction of the sequencing errors reassociates the two peptides to give a single protein, accounting for their appearance at a single spot.

As ICDSs (corresponding to authentic events or to sequencing errors) accounted for 1.4% of the ORF content *of M. smegmatis *mc^2^155, we surveyed a fraction of the proteome to determine the percentage of proteins originating from ORFs not predicted due to misannotations. We carried out two-dimensional electrophoresis of a soluble protein extract. The major spots (120) were excised, digested and analyzed by nano-LC-MS-MS (nanoflow liquid chromatography coupled to tandem mass spectrometry). We were able to identify about 250 proteins unambiguously by comparing the MS-MS data obtained from the tryptic peptides. We compared these MS-MS data directly with public nucleotide sequences, rather than using the classic comparison of MS-MS data with protein sequences [15,16] to prevent the introduction of bias. The identification of several proteins for a single spot is not surprising and has been widely reported in proteomic analysis [17]. For four spots the tryptic peptides identified by nano-LC-MS-MS analysis matched two contiguous hypothetical ORFs each (Table 3, Figure 2). There are two possible explanations for this finding. Firstly, two different proteins, encoded by two different frames in the same genome region, may be present in the same two-dimensional gel electrophoresis spot. This is unlikely, due to differences in molecular masses (Table 3), but cannot be entirely excluded. Secondly, these peptides may be derived from the same protein. In this case, a bypassed stop codon or a sequencing error could account for such an observation.

For the four proteins concerned, MS-BLAST showed that all the tryptic peptides identified matched the same protein on the basis of sequence similarity with other organisms. We carried out a new search with the MS-MS data obtained for the four two-dimensional gel electrophoresis spots using the corrected sequences obtained after resequencing of all the ICDSs. For all four spots the peptides were found to match in the same frame and new peptides from the proteins were detected (Table 3, Figure 2). We can conclude, therefore, that the four ICDSs detected were due to sequencing errors. These ICDSs are ICDS0019, ICDS0039, ICDS0040 and ICDS0093. We show ICDS0040 as an example in Figure 2.

Thus, proteome analysis identified errors in sequences that were not predicted to correspond to an ORF. All four cases detected in this way were found to correspond to sequencing errors (Table 1). There is, therefore, strong congruence between *in silico *data and nucleotide and proteomic analyses.

## Discussion

Previous *in silico *analyses have shown that all bacterial species contain ICDSs in their genome [5]. Here, using *M. smegmatis *and two experimentally independent approaches, we show that these ICDSs correspond to authentic mutations and to sequencing errors. By contrast, a recent large-scale proteome analysis (more than 900 proteins) of *M. smegmatis *mc^2^155 provided no evidence of sequencing errors [18]. Statistically, 16 sequencing errors should have been detected. Possible explanations for this discrepancy are that, by chance, no protein corresponding to an ICDS was extracted, or that proteins in conflict with genomic data were excluded from the analysis.

True frameshifts provide positive information, useful for characterization of the variation of amino acid sequences between various orthologs, whereas sequencing errors introduce noise and create artifactual genetic differences between strains and species. These sequencing errors may result from under-representation of the region in the genomic library or structures making sequencing difficult. Although most genomes have been sequenced with eight-fold coverage (each nucleotide being sequenced eight times), the sequences generated remain a statistical estimation and many regions of low coverage (less than three-fold) still exist in genome sequences [19]. No assembly data are available for the *M. smegmatis *genome project, but the sequencing errors are probably located in such low-coverage regions. In *M. smegmatis *mc^2^155, 28 of the 73 re-sequenced ICDSs were shown to result from errors. The correction of these errors modified the predicted amino acid sequences of the corresponding proteins. These changes in amino acid sequence increased similarity to orthologs, with consequences for comparative genomics. Unfortunately, it was not possible to associate a particular sequence or stretch of nucleotides with sequence errors. It is, therefore, not possible to predict whether a given ICDS corresponds to an authentic event or to a sequence error. The nature of each ICDS must, therefore, be investigated individually.

Modern biology approaches based on massive sequence comparisons need accurate sequences for meaningful analyses of genetic differences and similarities. Re-sequencing and the correction of errors in genomic sequences are likely to lead to the identification of new protein sequences. For instance, in *M. leprae*, which has a large number of ICDSs in its genome (845), even a small proportion of sequencing errors will provide researchers with substantial numbers of new protein sequences, making it possible to identify new functional genes, or to develop new serological tests.

Other mycobacterial species also contain ICDSs in their genome, some of which have been shown to correspond to authentic mutations acquired during evolution. For instance, the genomes of *M. tuberculosis *H37Rv, *M. tuberculosis *CDC1551 and *M. bovis *contain 96, 123 and 111 ICDSs, respectively, corresponding to about 2% of total gene content in each case [5]. Interestingly, a number of ICDSs corresponding to authentic events have been fortuitously characterized. In several cases it has been shown that these events inactivate the gene. For instance, ICDS0066 of *M. tuberculosis *H37Rv, corresponding to a gene encoding a polyketide synthase (*pks1*), includes a frameshift, generating two distinct ORFs, *pks1 *and *pks15*. In contrast, *M. bovis *and *M. leprae *carry a *pks1 *gene with no frameshift. The complementation of *M. tuberculosis *with the *pks1 *of *M. bovis *leads to the synthesis of a new metabolite, phenolphthiocerol [20]. Thus, *M. tuberculosis *has clearly lost the ability to synthesize phenolphthiocerol due to a frameshift within the *pks1 *gene. Another example is ICDS0067 in *M. bovis*, which occurs in a sequence encoding a putative glycosyltransferase. The ortholog of this gene has no frameshift in *M. tuberculosis *(Rv2958) [21]. The complementation of *M. bovis *BCG with Rv2958 from *M. tuberculosis *leads to the accumulation of a new product in this strain: diglycosylated phenolglycolipid [21]. Thus, *M. bovis *has lost the ability to metabolize the diglycosylated phenolglycolipid due to the frameshift within the glycosyltransferase gene.

These two examples, taken from published work, illustrate that, as expected, a frameshift within ORF may lead to a loss of function. It should be noted that the genes for which function has been lost (such as *pks1 *or *Rv2958*) have been split into only two pieces and could, therefore, theoretically revert to the wild-type allele with ease. These genes containing frameshifts are in the process of becoming pseudogenes (pseudogenization) but need to acquire additional mutations before they are fixed, leading to an almost irreversible loss of function.

The conclusion of this work may be extended to most, if not all, bacterial genomes sequenced to date. These findings have major implications for comparative genomics. Firstly, the resolution of sequencing errors reduces protein variability, facilitating the precise definition of module composition and function. Secondly, as ICDSs corresponding to authentic mutations probably lead to a loss of protein function, the choice of strain or species is of particular importance for investigations of the function of a particular gene. Researchers should carefully consider their investment before creating mutants in these ORFs or producing the corresponding polypeptides. It should be noted that a small number of ORFs containing frameshifts may retain their function or even lead to the acquisition of a new function. It would be interesting to re-frame these ORFs to evaluate the impact on protein function.

We have shown here that 28 of the 73 ICDSs resulted from sequencing errors. It seems highly likely that all sequenced genomes contain ICDSs resulting from sequencing errors. The current ICDS database contains more than 6,600 ICDSs (in 120 genomes) awaiting characterization. In this study, we detected sequencing errors at a rate of 4 per megabase. The calculated number of ICDSs is obviously an underestimate of the reality as some events such as fusion or fission that maintain the correct frame are not detected by the algorithm used [5].

Very few articles have dealt with sequence fidelity. TIGR has reported an error rate for finished genomes of 1 in 88,000 nucleotides [22,23] whereas Weinstock [19] estimated that the frequency of error was between 10^-3 ^and 10^-5^. The frequency of errors clearly depends on the chemical system used and the research centers carrying out the sequencing work [24]. The development of error prediction programs has greatly helped to reduce the error rate [2-4]. However, as shown in this study, sequencing errors are clearly a persistent problem in genomic databases. The major problem is that the bioinformaticians who assemble genomes have, for years, discarded precious information about how all the individual sequence fragments align on the assembled chromosome. The only way to test the nature of the ICDSs is to re-sequence the fragment. The NCBI has recently developed the 'Assembly Archive', which stores records of both the way in which a particular assembly was constructed and alignments of any set of traces to a reference genome [25]. This resource makes it possible to determine whether an ICDS corresponds to a region of low coverage and to evaluate the quality of the raw data. It would clearly be easier to resolve the ICDSs in various genomes if all the sequencing centers made complete assembly data available.

## Materials and methods

### Bacterial strains

*M. smegmatis *mc^2^155 (ATCC700084) and *M. smegmatis *NRRL B-692 (Trevisan) Lehman and Neumann (ATCC607) were purchased from the American Type Culture Collection (Manassas, Virginia, USA).

### ICDS detection in *M. smegmatis *mc^2^155

The genome sequence of *M. smegmatis *mc^2^155 was taken from the TIGR website [12]. The ICDSs were detected using the method developed by Perrodou *et al*. [5].

### Primer design and sequence analysis

The primers used to sequence frameshifts were designed as previously described [5] using an optimized version of the CADO4MI program (Computed Assisted Design of Oligonucleotides for Microarray). It is a freeware (GNU General Public License) accessible online [26]. For each genome, sequencing primers are available online [27]. The chromosomal DNA of the mc^2^155 and ATCC607 strains of *M. smegmatis *used for PCR amplification was purified as previously described [28]. Pairs of primers were used for amplification with Pfu Turbo DNA polymerase (Stratagene, La Jolla, CA, USA). PCR samples were run on a 0.8% agarose gel and the fragments were excised from the gel and purified using the QIAquick Gel purification kit (Qiagen Chatsworth, CA, USA). The PCR fragments had a mean length of 300 base-pairs. Purified PCR fragments were used as templates in sequencing reactions with each primer used for PCR amplification. The nucleotide and inferred aminoacid sequences were analyzed with DNA Strider [29]. Three independent amplicons were sequenced for each ICDS.

### Protein extraction and two-dimensional gel electrophoresis

*M*. *smegmatis *strain mc^2^155 (1 liter) was grown in M9 minimal medium (Difco, Detroit, USA) for 5 days and then centrifuged. Bacterial pellets were used for two-dimensional electrophoresis. Unless otherwise specified, all chemicals were obtained from Sigma (St Louis, MO, USA). Dithiothreitol (DTT) and iodoacetamide were obtained from Fluka (Buchs, Switzerland). The pellet fraction was incubated with extraction buffer (50 mM Tris, pH7.5, 1 mM phenylmethylsulfonyl fluoride, 1 mM EDTA, 1 mM DTT, protease inhibitor mixture (complete from Roche, Basel, Switzerland)) for 45 minutes at 4°C. The mixture was sonicated for a few seconds and its protein concentration determined by Bradford assay. The solvent of the protein extract was evaporated off and the protein residue was suspended in rehydration buffer (8 M urea, 2 M thiourea, 4% 3- [(3-cholamidopropyl)dimethylammonio]-1-propanesulfonic acid, 0.5% Triton X-100, 1% DTT, 20 mM spermine, 2% Pharmalyte (Amersham Pharmacia Biotech, Piscataway, NJ, USA)). The sample was incubated for 30 minutes at 20°C and centrifuged at 15,000 rpm at 20°C.

Protein extract was run on a strip of gel of pH range 3 to 10 (Bio-Rad Laboratories, Hercules, CA, USA) for 15 h at 20°C under 50 V in a PROTEAN isoelectric focusing cell (Bio-Rad). Isoelectric focusing was carried out with several voltage steps: 1 h at 200 V, then 4 h at 1,000 V followed by 16 h at 5,000 V and finally 7 h at 500 V at 20°C. The strips were incubated for 30 minutes at 20°C in electrophoresis buffer (50 mM Tris-HCl, pH 8.8, 6 M urea, 30% (v/v) glycerol, 2% (w/v) SDS, and 1% DTT), followed by 30 minutes in the same buffer supplemented with 2.5% iodoacetamide. Electrophoresis in a gradient gel (5% to 20% acrylamide) on a PROTEAN II (Bio-Rad) apparatus at 5 mA for 1 h and 10 mA overnight was used as the second dimension. The gel was stained with Colloidal blue (G260, Sigma); 120 spots were selected by visual inspection and gel slices were excised with a Proteineer SP automated spot picker (Bruker Daltonics, Bremen, Germany) according to the manufacturer's instructions.

### Mass spectrometry

The two-dimensional gel spots were excised, washed, destained, reduced, alkylated and dehydrated for in-gel digestion of the proteins with an automated protein digestion system, MassPREP Station (Waters, Milford, MA, USA). The proteins were digested overnight at room temperature with trypsin. They were then extracted with 60% (v/v) acetonitrile in 5% (v/v) formic acid and then with 100% acetonitrile. The resulting peptide extracts were analyzed directly by nano-LC-MS-MS on an Agilent 1100 Series capillary LC system (Agilent Technologies, Palo Alto, USA) coupled to an HCT Ultra ion trap (Bruker Daltonics). This instrument was equipped with a nanospray ion source and chromatographic separation was carried out on reverse phase (RP) capillary columns (C18, 75 μm id, 15 cm length, Agilent Technologies) with a flow rate of 200 nl/minute. The voltage applied to the capillary cap was optimized to -2,000 V. MS-MS scanning mode was performed in the Ultra Scan resolution mode at a scan rate of 26,000 m/z per second. Eight scans were averaged to obtain an MS-MS mass spectrum. The complete system was fully controlled by Agilent ChemStation and EsuireControl (Bruker Daltonics) software. The generated peak-lists of fragments were used for public *M. smegmatis *genome database searches.

## Figures and Tables

**Table 1:** ICDSs shown by resequencing to correspond to sequencing errors in *M. smegmatis *mc^2^155

ICDS number	5' position	ORF number	Putative function	Functional classification	Accession number	Type of event
0012	1639371	1547	Hypothetical	Unknown	DQ866846	U
0019	1918521	1842-1843	Adenosylhomocysteinase	Intermediary metabolism	DQ866847	U
0022	1930746	1854-1855	Sodium/proton antiporter	Cell wall, process	DQ866848	U
0024	2055797	1975-1976	Methane/phenol/toluene hydroxylase	Intermediary metabolism	DQ866849	O
0026	2119141	2042	Conserved hypothetical	Unknown	DQ866850	O
0027	2162020	2086-2087	Ferredoxin-NADP reductase	Intermediary metabolism	DQ866851	O
0028	2221312	2149-2150	Hypothetical	Unknown	DQ866852	U
0030	2290855	2215-2216	CoA-transferase	Intermediary metabolism	DQ866853	O
0035	2799279	2732-2733	Conserved hypothetical	Unknown	DQ866854	U
0039	3216877	3151	Aconitate hydratase	Intermediary metabolism	DQ866855	O (× 2)
0040	3262835	3192-3193	Maltooligosyltrehalose synthase	Intermediary metabolism	DQ866856	U
0041	3313327	3240	ABC transporter (CydC)	Intermediary metabolism	DQ866857	O
0051	3902349	3837	Dephospho-CoA kinase	Intermediary metabolism	DQ866858	O (× 2)
0053	3961899	3892-3893	Transcriptional regulator	Regulation	DQ866859	O
0054	4017126	3952-3953	Hypothetical	Unknown	DQ866860	O
0057	4255762	4183	Pyruvate dehydrogenase	Intermediary metabolism	DQ866861	U
0058	4288648	4211-4212	Nitrate reductase	Intermediary metabolism	DQ866862	U
0061	4637174	4539-4540	Oxidoreductase	Intermediary metabolism	DQ866863	O
0072	5644787	5533-5534	Hypothetical	Unknown	DQ866864	U
0073	5855980	5754	Acetyltransferase	Intermediary metabolism	DQ866865	O
0076	6078397	5970-5971	Fatty-acid CoA synthetase	Lipid metabolism	DQ866866	U
0080	6600510	6504-6505	Conserved hypothetical	Unknown	DQ866867	U
0082	6670969	6579	Helicase	DNA metabolism	DQ866868	O
0083	6673489	6581	Hypothetical	Unknown	DQ866869	U
0089	342400	*	Methyltransferase	Intermediary metabolism	DQ866870	U
0091	601272	0511-0512	Hypothetical	Unknown	DQ866871	U
0092	809979	0716-0717	Transcriptional regulator	Regulation	DQ866872	U
0093	428949	1395-1396	Elongation factor G	Translation	DQ866873	O

The nucleotide position, the affected ORF (according to the TIGR website), its putative function computed after the correction of the sequencing errors, its functional classification and its accession number are indicated for each ICDS. The asterisk indicates an ORF not predicted by TIGR. Two types of error were observed: overcall (O), an extra nucleotide not present in the target sequence was initially predicted at a given position; and undercall (U), a nucleotide corresponding to a true target sequence was not predicted at a given position.

**Table 2:** ICDSs shown by resequencing to correspond to authentic mutations in both *M. smegmatis *mc^2^155 and ATCC607

ICDS number	5' position	ORF number	Putative function	Functional classification
0003	1169121	1094-1095	Oxidoreductase	Intermediary metabolism
0004	1232918	1164-1165	Arsenic resistance protein	Cell wall, process
0005	1277324	1200-1201	Glycosyltransferase	Intermediary metabolism
0006	1304141	1226-1227	ABC transporter (permease)	Cell wall, process
0007	1508649	1403-1404	Sodium/proton antiporter	Cell wall, process
0008	1510156	1405-1406	Arginine/ornithine antiporter	Cell wall, process
0009	1510156	1405-1407	Arginine/ornithine antiporter	Cell wall, process
0010	1510315	1406-1407	Arginine/ornithine antiporter	Cell wall, process
0011	1545509	1447	Secreted immunogenic protein (Mpt70)	Cell wall, process
0013	1645546	1552-1553	Conserved hypothetical	Unknown
0014	1650143	1557-1558	Hypothetical	Unknown
0015	1669043	1575-1576	Hypothetical	Unknown
0020	1922875	1848-1849	Formate dehydrogenase, alpha subunit	Intermediary metabolism
0021	1924487	1849	Formate dehydrogenase, alpha subunit	Intermediary metabolism
0023	2026072	1949-1950	Hypothetical	Unknown
0025	2097821	2019-2020	Cytochrome P450	Intermediary metabolism
0029	2234814	2164-2165	Substrate-CoA ligase	Lipid metabolism
0033	2557504	2472-2473	Sugar transporter	Cell wall, process
0036	2877071	2816-2817	Two-component system regulator	Cell wall, process
0038	3161135	3097-3098	O-methyltransferase	Intermediary metabolism
0042	3351460	3281-3282	Sugar ABC transporter	Cell wall, process
0043	3410192	3341	Fatty acid desaturase (DesA3)	Lipid metabolism
0044	3442071	3378	Dehydrogenase/reductase	Intermediary metabolism
0045	3471038	3405-3406	Hypothetical	Unknown
0046	3506575	3443-3344	Hypothetical	Unknown
0049	3849109	3785	Conserved hypothetical	Unknown
0052	3930423	3862-3863	Polyprenol-monophosphomannose synthase (Ppm1)	Cell wall, process
0055	4172910	4102-4103	Dehydrogenase	Intermediary metabolism
0059	4551995	4464-4465	Hypothetical	Unknown
0063	5113475	5001	Transporter	Cell wall, process
0064	5127828	5017-5018	Multidrug resistance efflux protein (Tap)	Cell wall, process
0067	5238606	5122-5123	Nitrate reductase (NarX)	Intermediary metabolism
0070	5596138	5488	Conserved hypothetical	Unknown
0071	5639815	5527-5528	Protein-glutamate methylesterase	Intermediary metabolism
0074	6014123	5909-5910	Hypothetical	Unknown
0075	6071755	5963-5964	Integral membrane protein	Unknown
0078	6147983	6046	AraC-family transcriptional regulator	Regulation
0079	6260084	6152-6153	Anion transporter	Cell wall, process
0084	6846273	6761	Oxidoreductase	Intermediary metabolism
0085	6862121	6775	Major facilitator transporter	Cell wall, process
0086	6955671	6870-6871	Glutamine transporter	Cell wall, process
0087	6977889	6889-6890	Thioredoxin	Intermediary metabolism
0088	17247	0017-0018	Hypothetical	Unknown
0094	3456823	*	Dihydrolipoamide dehydrogenase	Intermediary metabolism

The nucleotide position, the affected ORF (according to the TIGR website), its putative function and its functional classification are indicated for each ICDS. The asterisk indicates an ORF not predicted by TIGR.

**Table 3:** ICDSs shown by nano-LC-MS-MS analysis to correspond to sequencing errors in *M. smegmatis *mc^2^155

ICDS number	Affected ORF	Calculated mass before correction	Calculated mass after correction
0019	1842-1843	45,980-7,370	53,460
0039	3151	64,570	101,200
0040	3192-3193	48,730-33,880	83,490
0093	1395-1396	21,560-63,800	77,220

The affected ORFs (according to the TIGR website) and their predicted molecular weights before and after genomic correction are indicated.
